# Supplementary figures and images for: Disruption of the mRNA m6A writer complex triggers autoimmunity in Arabidopsis
Source: PLoS Genet. 2025 Nov 6;21(11):e1011925. doi: 10.1371/journal.pgen.1011925 (PMC12617952; doi:10.1371/journal.pgen.1011925)

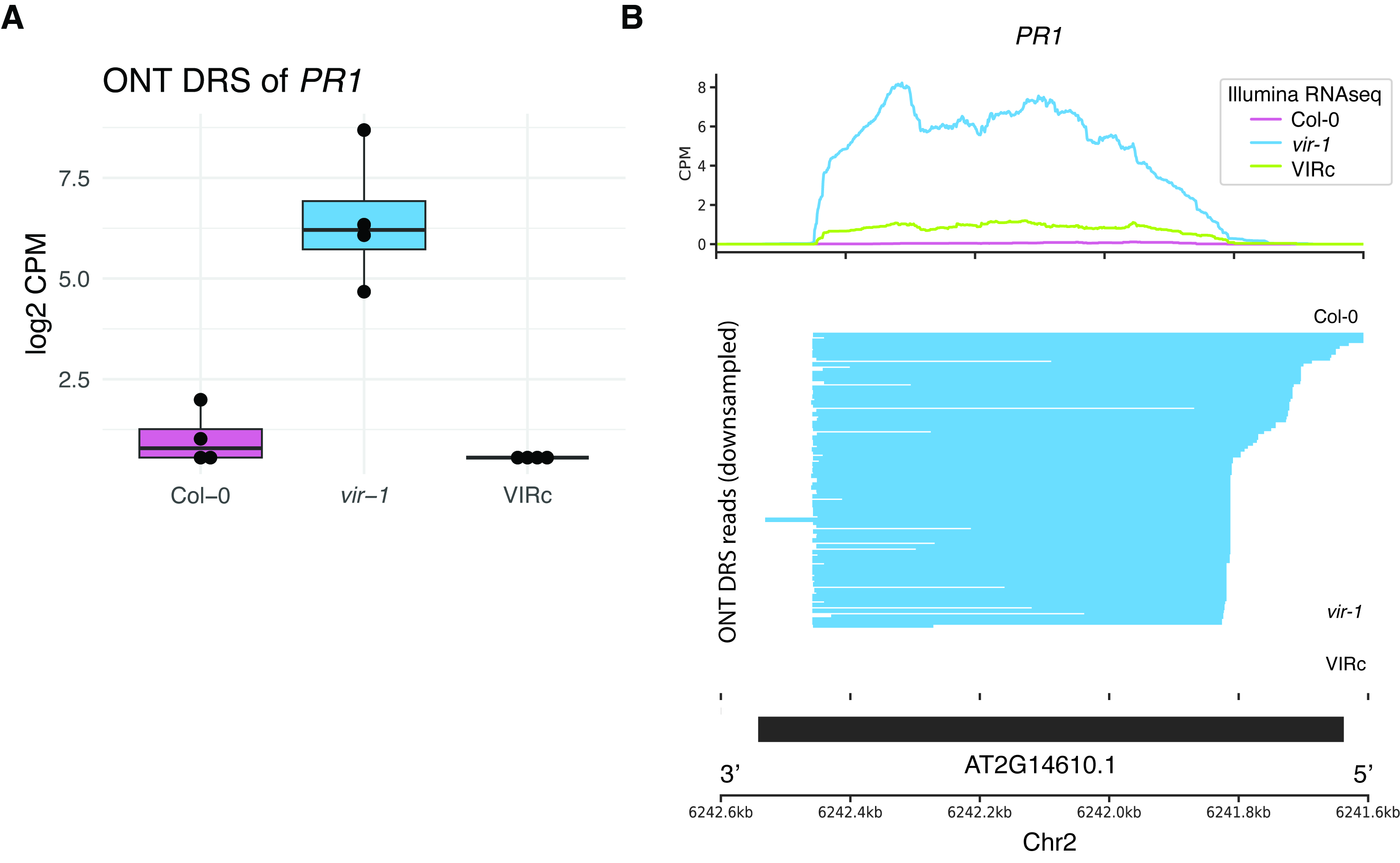

Supplement: S1 Fig — A) Normalised log2 counts per million of PR1 (AT2G14610) in Col-0, Col-0, vir-1 and VIRc in ONT DRS reads (n = 4 samples per genotype). B) Upregulation of PR1 (AT2G14610) in vir-1 at 20°C, shown by a gene track of Illumina RNA-seq and downsampled ONT DRS reads. (TIF) [file pgen.1011925.s001.tif]

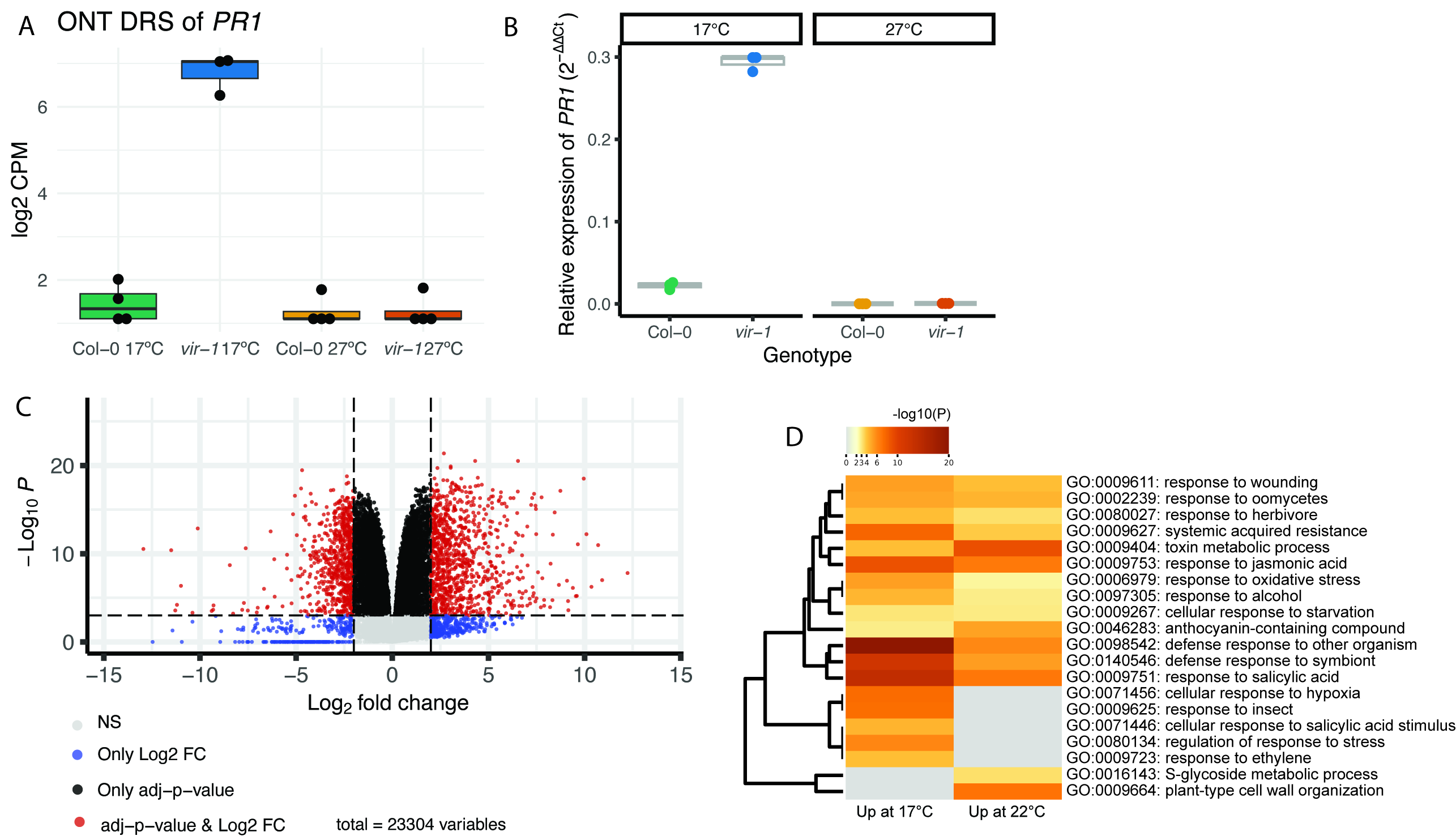

Supplement: S2 Fig — B) RT-qPCR showing the upregulation of PR1 in vir-1 at 17°C (n = 3 per condition). C) Volcano plot showing the log2 fold change and adjusted p-value of differential gene expression in vir-1 at 17°C contrasted to the average expression in vir-1 at 27°C and Col-0 at 17°C and 27°C. Genes with log2FC > 2 and p < 0.001 are coloured in red, genes which only pass the p-value threshold are coloured in black, and genes which only pass the log2FC threshold are coloured in blue. Non-significant changes (NS) are coloured in grey. Source data available in S5 File. D) Overlap in enriched GO terms between genes upregulated at 17°C contrasted to the average expression in vir-1 at 27°C and Col-0 at 17°C and 27°C, and genes which were significantly upregulated in vir-1 at 22ºC contrasted to Col-0 at 22ºC. (TIF) [file pgen.1011925.s002.tif]

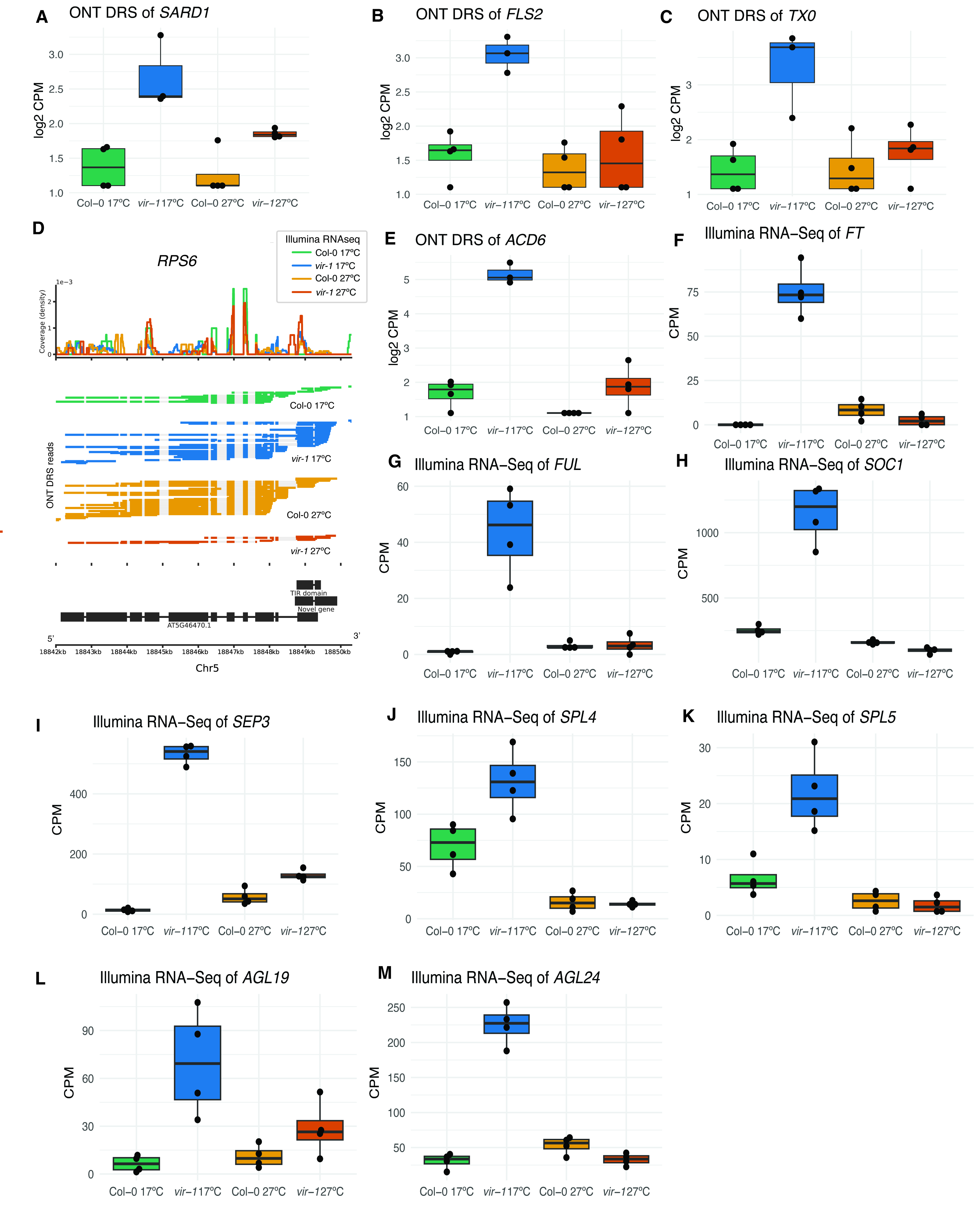

Supplement: S3 Fig — B) Upregulation of FLS2 (AT5G46330) in vir-1 at 17°C, shown by a boxplot of normalised expression (log 2 counts per million) in ONT DRS data (n = 3–4 samples per condition). C) Upregulation of TX10 (AT1G57630) in vir-1 at 17°C, shown by a boxplot of normalised expression (log 2 counts per million) in ONT DRS data (n = 3–4 samples per condition). D) Gene track of ONT DRS data showing the upregulation of a novel TIR domain-containing gene (annotated as Novel gene) downstream of RPS6 (AT5G46470) in vir-1 at 17°C (n = 3–4 samples per condition). E) Upregulation of ACD6 (AT4G14400) in vir-1 at 17°C, shown by a boxplot of normalised expression (log 2 counts per million) in ONT DRS data (n = 4 samples per condition). F-M) Boxplots showing the normalised log 2 counts per million (as produced by edgeR) for the flowering genes; FT (AT1G65480), FUL (AT5G60910), SOC1 (AT2G45660), SEP3 (AT1G24260), SPL4 (AT1G53160), SPL5 (AT3G15270), AGL19 (AT4G22950) and AGL24 (AT4G24540), in Illumina RNA-seq of vir-1 and Col-0 at 17ºC and 27ºC (n = 4 samples per condition). (TIF) [file pgen.1011925.s003.tif]

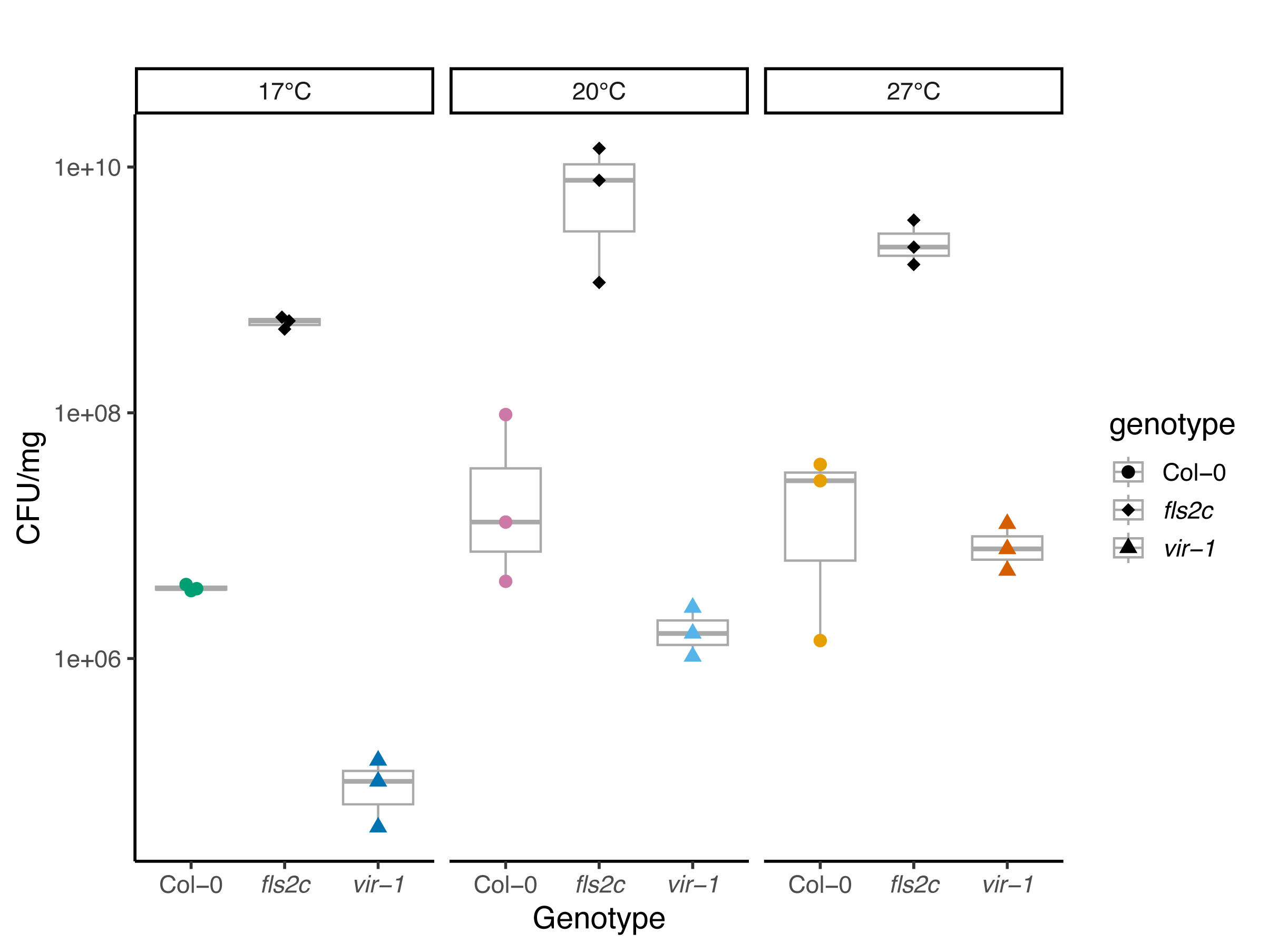

Supplement: S4 Fig — Bacterial populations were quantified at 3 days post-inoculation (dpi) (n = 3 per condition). One way ANOVA tests on each genotype revealed a significant effect of temperature in the vir-1 genotype (F = 23.02, p = 0.00197) which was not present in Col-0 WT or fls2. Source data available in S9 File. This experimental analysis represents an independent replication of the experiment presented in Fig 4A. (TIF) [file pgen.1011925.s004.tif]
