# Supplementary material for: Disruption of the mRNA m6A writer complex triggers autoimmunity in Arabidopsis
Source: PLoS Genet. 2025 Nov 6;21(11):e1011925. doi: 10.1371/journal.pgen.1011925 (PMC12617952; doi:10.1371/journal.pgen.1011925)
Supplement: Attachment — Submitted filename: 155428_1_rebuttal_3426220_sy5f66.pdf [file pgen.1011925.s024.pdf]

Full Revision

Review COMMONS

Manuscript number: RC-2025-0219
Corresponding author(s): Gordon, Simpson

[Please use this template only if the submitted manuscript should be considered by the affiliate journal as a full revision in response to the points raised by the reviewers.

If you wish to submit a preliminary revision with a revision plan, please use our "Revision Plan" template. ***It is important to use the appropriate template to clearly inform the editors of your intentions.****]*

1. General Statements [optional]

This section is optional. Insert here any general statements you wish to make about the goal of the study or about the reviews.

2. Point-by-point description of the revisions

This section is mandatory. Please insert a point-by-point reply describing the revisions that were already carried out and included in the transferred manuscript.

Response to Reviewers' Comments

We are grateful to the reviewers for taking the time to review our manuscript. We address the reviewers' comments in the order in which they were presented to us and respond to each comment below, as indicated by blue-coloured text.

Reviewer 1
Reviewer #1 (Evidence, reproducibility and clarity (Required)):

Summary
The authors aim to understand the consequences of disrupting N6 methyladenosine, an abundant mRNA modification in plants and other organisms, in Arabidopsis. Genetic ablation of the N6 methyladenosine transferase complex is embryonic lethal in Arabidopsis. Therefore, the authors utilize a hypomorphic allele of VIRILIZER, a component of the complex, to examine gene expression changes and other phenotypes. The authors demonstrate that immune response pathway genes are misregulated in the vir mutant. This transcriptional phenotype is suppressed at higher temperatures, although developmental phenotypes are not. The manuscript provides strong evidence that reduced function of the m6A methyltransferase complex leads to upregulation of immune response genes, although a mechanistic connection between the immune response and m6A in mRNA is not discerned.

Major comments
The major claims of the manuscript are that disrupting the m6A writer complex triggers an autoimmune response that is present at 17C and suppressed at 27C (in line with known aspects of Arabidopsis immunity). Consistent with this, they also show that at 17C the vir-1 mutant has more cell death and is more resistant to infection by Pseudomonas syringae. All of these claims are well supported by the data. The authors also claim that polyA tail lengths are different between the two temperatures. They further speculate that mRNAs that lack m6A trigger immune signaling, but this is not directly tested in the study.

The conclusions about transcriptional activation of the immune response at lower temperatures are sufficiently supported by two types of mRNA sequencing data (direct RNA sequencing and short-read sequences) and appropriate biological replication. The initial profiling was at 22 C, later profiling was at 17C and 27 C. How similar/overlapping were the vir-1 misregulated genes at 17C and 22C? Is the immune response transcriptional signature stronger at 17C than at 22C?

Yes, the immune response transcriptional signature appears to be stronger at 17 °C than at 22 °C: Of the 1,215 genes with significantly increased expression in *vir-1* mutants at 17 °C (compared to Col-0 at 17 °C, using a threshold of adjusted $p < 0.001$ and $\log_2 FC > 2.0$), only 219 genes were also upregulated in *vir-1* at 22 °C. Of the 976 genes with significantly decreased expression in *vir-1* mutants at 17°C (compared to Col-0 at 17 °C, $p < 0.001$ and $\log_2 FC < -2.0$), only 48 genes (3.8%) were also downregulated in *vir-1* at 22 °C. 123 genes with 'defense' or 'defence' included in their TAIR description were significantly upregulated at 17 °C compared to 71 genes at 22 °C. 65 genes with 'defense' or 'defence' included in their TAIR description were significantly downregulated at 17 °C compared to 9 at 22 °C.

It is important to note that since the two RNA-seq experiments were performed separately, the results are not directly comparable, and hence this comparison is not included in our manuscript. Nonetheless, this conclusion aligns with the differential susceptibility to *P. syringae* infection shown in Figure 4A and Supplementary Figure 4 of our revised manuscript. In these pathogen infection analyses, which were conducted side by side, increased bacterial colony counts were measured on *vir-1* mutants at 21 °C compared to 17 °C indicative of a stronger immune response at 17 °C.

The authors sought to determine whether the vir-1 response at 17C was due to pathogen infection of those plants. They used their Illumina RNA-seq data to try and identify pathogen RNAs. They report that there was no significant enrichment of plant pathogen sequences (supplemental table 7). Significant compared to what? Supplemental Table 7 does not indicate that the WT data was assessed and there's no information on significance of enrichment (or nothing obvious, based on column titles). Did the Illumina library prep preparation rely on polyA tails? If so, this is not a sensitive assay to detect bacterial transcripts.

We attempted to determine whether the observed defence response was due to contamination in the mutant samples caused by the presence of a pathogen or reflected an autoimmune response. To address this question, all mutant RNA-seq Illumina data from 17 °C and 21 °C were subjected to a single *de novo* RNA-seq assembly after host (Arabidopsis) removal. The resulting single transcriptome assembly was then used to map all the sequencing data from mutant and Col-0 (wild type) to obtain digital quantification per transcript per condition per replicate.

We know there are many differentially expressed genes and transcripts between the two conditions (as the manuscript shows). Therefore, we narrowed our search by annotating the transcriptome assembly using DIAMOND-BLASTX vs GeneBank NR (e-value 1e-15), to return a list of hits and the putative origin of the transcript. There were 23 transcripts, of which the top Diamond-BLASTX hit was to a potential plant pathogen. The resulting "putative pathogen" counts per transcript were then used for differential transcriptome expression analysis: mutant vs Col-0 (per temperature). There was no significant difference between the WT Col-0 and *vir-1* mutant RNA-seq putative pathogen transcripts, at a logFC threshold of 2.0 and FDR 0.001.

In the revised manuscript, we have included the normalised expression profile of counts for the potential pathogen reads in a tab of Supplementary Table 7 (note the low abundance of many of these) and added new text to the Results section to clarify what we mean by significant.

We acknowledge that this analysis has limitations. As the reviewer notes, since the Illumina libraries were prepared using poly(A) selection, this approach is unlikely to capture bacterial transcripts comprehensively. However, fungal and oomycete pathogens would be detected, as would oligoadenylated bacterial transcripts.

We have added new text to this section in the revised manuscript to acknowledge the limitations of this analysis. For transparency, we have retained this section in the Results, because to the best of our knowledge, this type of control is not routinely included in Arabidopsis autoimmunity analyses.

I found the last section on altered poly(A) tail length and site usage somewhat difficult to follow and the analysis rather cursory.

We apologise if this is the impression given by this section. Like the reviewer, we were intrigued by the findings on poly(A) tail length and have asked ourselves many of the same questions that the reviewer poses. However, we decided that the major conceptual advance we were making here related to autoimmunity and its implications for understanding the impact of m^6A. We cannot yet conclude whether changes in poly(A) tail length are a cause or consequence of immune activation or the interplay between this and the absence of m^6A writer components. Consequently, we found that too much focus on poly(A) tail length detracted from the major conceptual advance of the manuscript. Nevertheless, the poly(A) tail phenotypes are so clear that it is important to report them. Furthermore, as ONT DRS makes estimates of poly(A) tail length data readily available, the field as a whole can begin to reveal how poly(A) tail length changes arise in m^6A writer complex mutants. We have addressed the specific comments made by the reviewer to improve this section in the revised manuscript (see below).

The authors find no difference in polyA site usage in vir-1 at 17C or 27C (although both are different than WT). For Figure 7A, in addition to the histogram of poly A site shift, I would like to see a plot (heatmap?) that compares poly A sites shift for individual mRNAs across samples, instead of only aggregated data.

In response to the reviewer's request, we provide an analysis of the shift in poly(A) site usage at an individual gene across samples using *ISTL* (AT1G25420) as an example. For transparency, we present this analysis based on total 3' end read counts (A) and as a percentage of total 3' end read counts (B) mapped to this gene. In this example, a single distal site is preferentially used in wild-type Col-0 (indicated by a black arrow). In contrast, two promoter-proximal poly(A) sites are used preferentially in *vir-1* (indicated by red arrows), and the distal site, which was preferentially used in Col-0, is almost unused in *vir-1* (Response Figure 1). Therefore, consistent with the aggregated data, poly(A) site shifts are evident between genotypes, rather than between temperature treatments, at individual genes.

We have now included this figure as panel B of Figure 7 in the revised manuscript, along with new text that describes it.

Review COMMONS

A

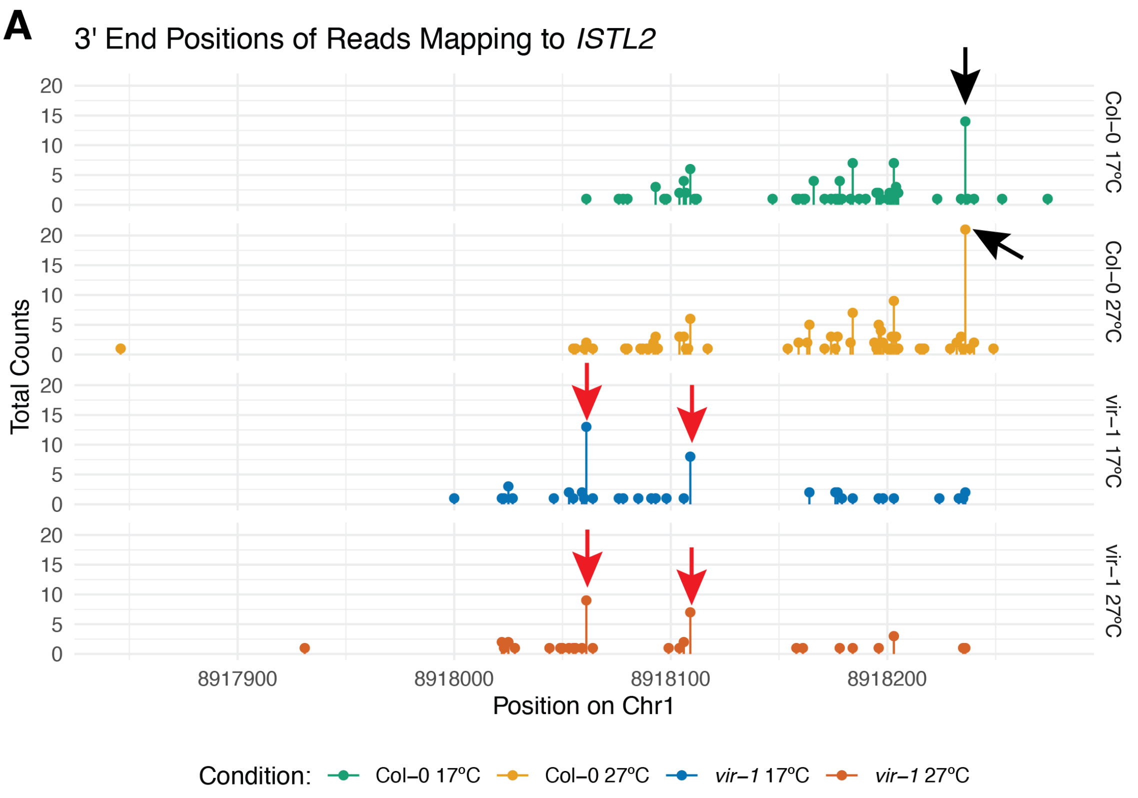

B

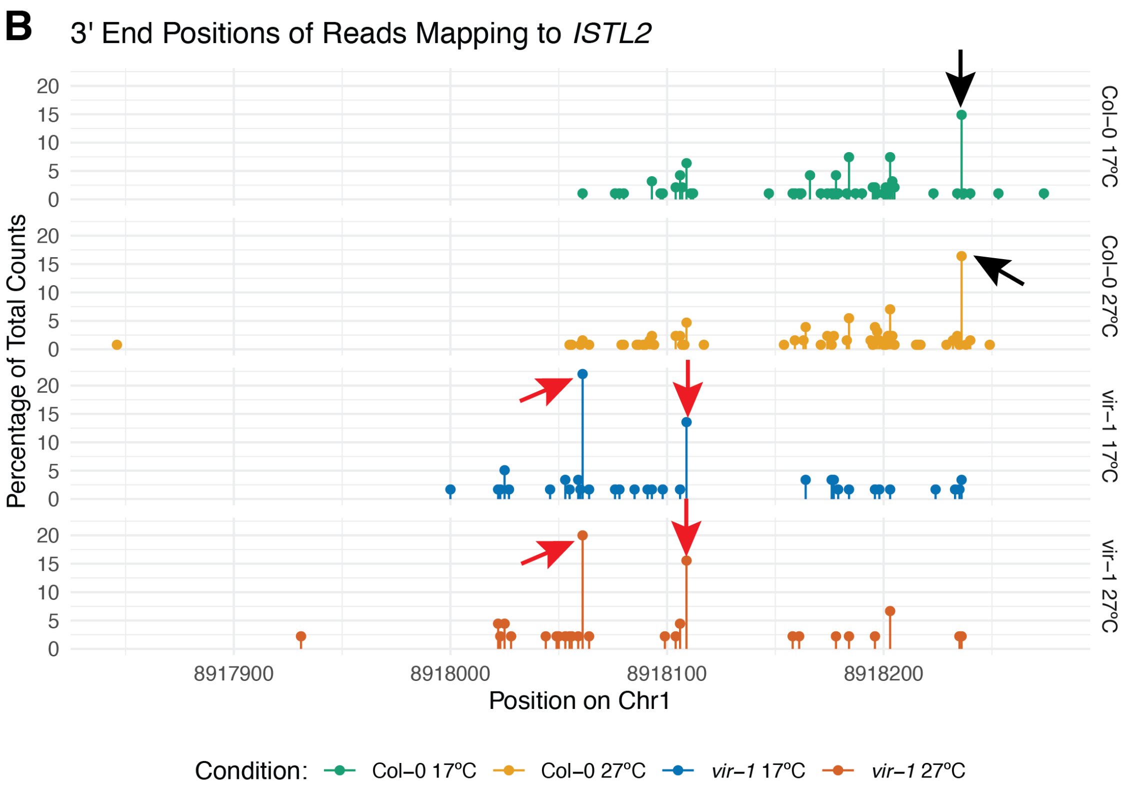

Response Figure 1: Position of the end of reads mapping to the 3' end of *ISTL2*, plotted as A) the read count and B) the percentage of the total number of reads in the condition. The position of the major distal poly(A) site in use in Col-0 is indicated by a black arrow, and the proximal sites with increased usage in *vir-1* are indicated with red arrows.

Are there individual mRNAs that differ between 17 and 27C in vir-1?

Yes, but the number is relatively low. Only 30 genes with a significant difference in poly(A) site usage between *vir-1* mutants at 17°C and *vir-1* mutants at 27°C were detected. This is comparable to the 48 genes with a significant difference in poly(A) site usage between wild-type Col-0 at 17 °C and wild-type Col-0 at 27 °C. In contrast, we identified 981 genes with significant differences in poly(A) site usage between Col-0 and *vir-1* at 17 °C and 820 genes with significant differences in poly(A) site usage between Col-0 and *vir-1* at 27 °C.

We have included these details in the revised manuscript.

A similar comment applies to the data in 7E. Please also compare individual mRNA polyA tail length across samples.

In response to the reviewer's request, we assessed poly(A) tail length distributions for *GAPC2* (AT1G13440) and *UBQ10* (AT4G05320) transcripts, because they are highly expressed and stably detected across the experimental conditions analysed here. These genes display tail length distribution patterns that closely mirror the global read poly(A) tail length estimates observed across temperatures and genotypes: fewer short poly(A) tails and more long poly(A) tails detected in *vir-1* than Col-0 at 17°C and more short poly(A) tails and fewer long poly(A) tails detected in *vir-1* than Col-0 at 27°C (Supplementary Figure 7C-D). In a previous study (Parker et al., 2020), we reported changes in poly(A) tail length distribution of *CAB1* (AT1G29930) transcripts between wild-type Col-0 and *vir-1* mutants. In the current dataset, *CAB1* also exhibits temperature- and genotype-dependent shifts in tail length: fewer short poly(A) tails and more long poly(A) tails were detected in *vir-1* than Col-0 at 17°C (Supplementary Figure 7B). However, at 27°C more of the shortest poly(A) tail length distributions are detected in Col-0. suggesting a degree of gene-specific variation.

We have included these details in the revised manuscript.

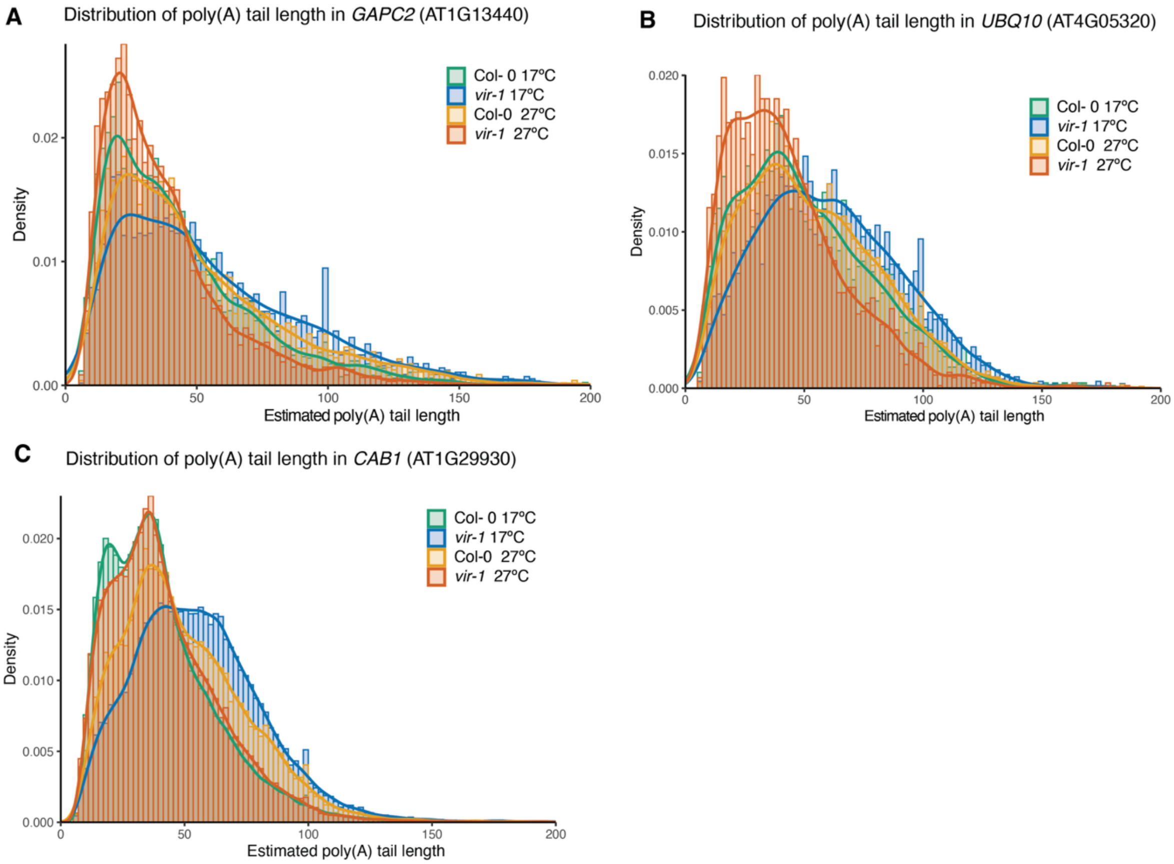

Response Figure 2: Distribution of poly(A) tail lengths for A) *GAPC2* (AT1G13440) and B) *UBQ10* (AT4G05320), C) *CAB1* (AT1G29930), in Col-0 and *vir-1* in 17ºC and 27ºC.

What is the significance of the change in polyA tail length? The tails are shorter in vir-1 than Col at 27 C. But vir-1 has a very similar phenotype to WT at 27 C. At 17 C, vir-1 tails are longer than WT. Together, do these two results imply that polyA tail length is unlikely to be related to the observed phenotypes? In other words, if longer tails have no effect, do shorter tails?

To clarify, although many of the gene expression differences observed in *vir-1* at 17 °C return closer to wild-type Col-0 levels at 27 °C, *vir-1* mutants still exhibit distinct developmental differences from Col-0 under both conditions.

It remains unclear if the observed changes in poly(A) tail length are causally linked to the autoimmune phenotype. Several possibilities exist: (i) immune activation may alter poly(A) tail processing; (ii) changes in poly(A) tail length may contribute to triggering an immune response; or (iii) both phenomena may occur independently as downstream consequences of disrupting the m^{6}A writer complex in *vir-1* mutants. These conclusions are consistent with the Discussion section in the revised manuscript.

Is there any relationship between RNAs with altered polyA site usage or tail length and those mRNAs that are misexpressed in the mutant?

Many of the transcripts that are significantly upregulated in the *vir-1* mutant at 17ºC are expressed at low levels (or not at all) in the other conditions. This presents a challenge when comparing poly(A) site choice and tail length distribution between conditions.

For the transcripts upregulated in *vir-1*, which were expressed in the other conditions, the poly(A) tail distribution resembled the global distribution, with longer median poly(A) tails in *vir-1* at 17 °C and shorter median poly(A) tails in *vir-1* at 27 °C. In other words, the poly(A) tail phenotypes we report here are not limited to the immune response genes upregulated in *vir-1* at 17 °C. A plot demonstrating this has been added to Supplementary Figure 7.

According to a Fisher's exact test, genes with significantly altered poly(A) site usage were not enriched among those significantly misexpressed (FDR < 0.001) in *vir-1* at 17ºC compared to other conditions, and there was no correlation between gene expression and the changes in poly(A) site usage observed between Col-0 and *vir-1* at either temperature.

We have added text to the Results section to incorporate this detail into the revised manuscript.

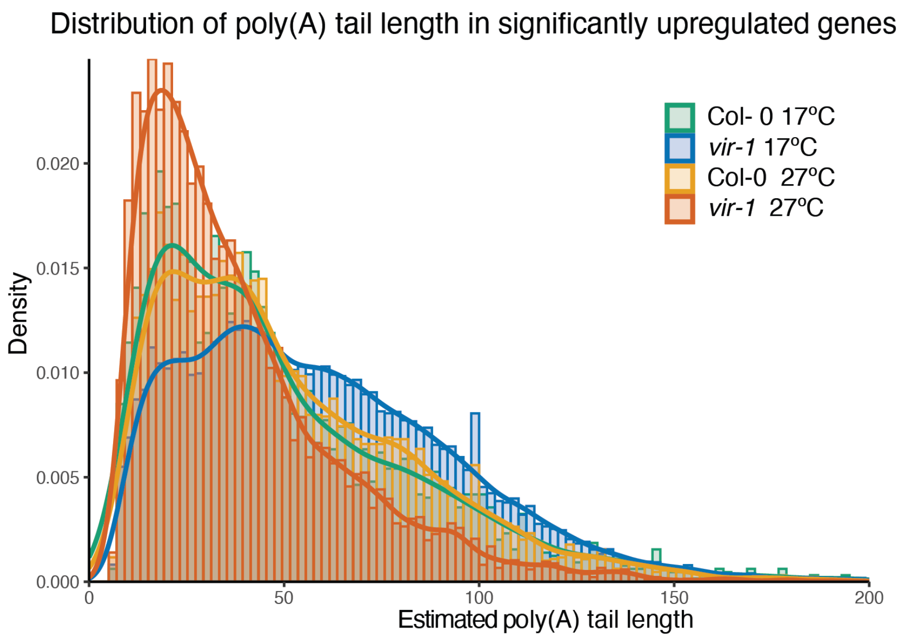

Response Figure 5: Distribution of poly(A) tail length estimates for genes with significantly increased expression in *vir-1* at 17 ºC compared to other conditions.

Are immunity gene mRNAs more likely to be m6A modified than other mRNAs?

No, for the transcripts with sufficient sequencing coverage, we did not observe any enrichment of m^{6}A sites among immunity-related mRNAs compared to the transcriptome as a whole. It should

be noted, however, that most immunity-related transcripts are expressed at low levels in wild-type Col-0 plants in the absence of pathogen infection. Such low expression levels pose a technical limitation on our ability to assess their m^6A modification status, as *m6Anet* requires a minimum coverage of 20 reads per site to reliably predict modifications. However, for the transcripts with sufficient coverage (greater than 20 mean reads in Col-0 across 17ºC and 27ºC treatments), we observe 41% fewer m^6A sites among genes with TAIR annotations including the terms 'defense' or 'defence' compared to the Col-0 transcriptomes as a whole (Fisher's exact test: $p = 6.24 \times 10^{-6}$, odds ratio = 0.59).

We have added this clarification to the text in the revised manuscript.

Minor Comments

At times it felt like the authors were stretching to fill seven figure with data. For example, in Figure 1, it was not necessary to show the data on increased PR1 expression in 6 different sub panels (B-F) to convince the reader that PR1 expression was increased.

Figure 1 has been revised to include only panels A-C and F; with panels D and E moved to a new Supplementary Figure 1.

A similar comment applies to Figure 3A-D. In Figure 3 please write the common gene names above the plots.

Figure 3, and other figures that feature logged counts per million plots, have been updated to include common gene names in plot titles rather than y-axis labels.

In Supplemental Table 3 the Enriched GO Terms tab is blank.

We thank the reviewer for bringing this to our attention. Table 3 has now been updated to include the enriched GO terms.

Supplementary File 1 appeared to be missing from the submission, so I could not evaluate the sequencing statistics (# of reads per sample, mapping %, etc).

We apologise for this oversight. We added Supplementary File 1 to the publicly available Zenodo repository alongside our preprint when this was brought to our attention. We have also uploaded Supplementary File 1 alongside the revised manuscript.

Many of the Supplemental Tables would benefit from a readme that describes what analysis was performed and what the different columns mean.

We are grateful for this suggestion. We have updated our supplementary tables to include a README and more informative file names. Supplementary Table numbering has been updated to include Supplementary File 1 as Supplementary Table, with the original Supplementary Tables 1 and 2 combined.

Reviewer #1 (Significance (Required)):

The manuscript provides additional insight on the functional consequences of disrupting adenosine methylation in RNA, identifying features of an autoimmune response. Given the ubiquity of m6A in RNA across eukaryotes, this is a result that will be of interest to basic researchers in the plant RNA modification community and likely those working in other eukaryotes. However, the study is not able to connect the inappropriate expression of immune response genes back to the function m6A in RNA, and the effects might be indirect. Although there is speculation that RNA that lacks m6A might trigger autoimmunity, the presented experiments do not directly test that hypothesis (nor do the authors claim to).

Reviewer 2
Reviewer #2 (Evidence, reproducibility and clarity (Required)):

The manuscript by Metheringham et al. reports on interesting new characterizations of phenotypes caused by genetic inactivation of subunits of the methyl transferase complex responsible for N6-adenosine methylation in (pre)-mRNA ("the m6A writer") in the plant Arabidopsis thaliana. The main claim of the paper is that mutants in these subunits exhibit autoimmunity, a claim that is supported by the following lines of evidence:

• Transcriptome profiling by mRNA-seq shows a gene expression profile with differential expression of many stress- and defense-related genes.
• The immunity-like gene expression profile is observed under growth at 17{degree sign}C but not at 27{degree sign}C, consistent with the well-known temperature-sensitivity of some (but not all) innate immunity signaling systems in plants.
• m6A writer mutants show increased resistance to infection by the virulent Pseudomonas syringae DC3000 strain.
• The primary biochemical defect in m6A writing is not temperature sensitive, excluding the trivial possibility that the mutant alleles chosen for study are simply ts.

The observations are important and the manuscript is very well written, a pleasure to read: the problem is clearly presented, the experimental results are presented in a clear, logical succession, and the discussion treats important points.

The study is valuable pending some manuscript revision on the autoimmunity interpretation of the results obtained, and a few suggested edits that can be included if the authors agree that they would improve the paper.

The finding that an autoimmune-like state is activated in m6A writer mutants is significant because it provides a warning flag on how such mutants should be used for studying the role of m6A in stress response signaling, including reassessment of previously published work. Whether the stress state really is autoimmunity is subject to some debate, particularly because no genetic evidence to support it has been obtained. The results are nonetheless interesting and constitute an important contribution to the community, even if they remain descriptive and with nearly no insight into molecular mechanisms. My suggestions for improvement are summarized below.

(1) Although the authors do a lot to support the claim that autoimmunity is an element of m6A writer mutant phenotypes, the study does not include genetic evidence to support this claim. This is important, because if the stress/defense gene activation causes some of the morphological phenotypes of m6A writer mutants, one should be able to suppress such defects by mutation of know immune signaling components such as the appropriate nucleotide-binding leucine-rich repeat proteins, or more generic signaling components such as EDS1, PAD4 and SAG1, common

to a subset of such intracellular immune receptors. Resistance to pathogens can be observed in mutants with constitutive stress response signaling, and defense-like gene expression can be induced as a secondary of other primary defects, for instance DNA damage. Similarly, while it is true that some types of immune activation are temperature sensitive, others are not 1, and clearly, elevated temperature changes so much of the physiology of the plant that sensitivity to elevated temperature cannot be used as proof of immune activation. Thus, each of the lines of evidence presented is suggestive, not conclusive. Together, they constitute a good argument, but still not a completely satisfactory proof of the main claim.

I do not think that this concern means that a lot of genetic work must be undertaken to make this paper publishable, but I think that the authors should be even more careful about how they interpret their observations. I understand that they favor more or less direct activation of autoimmunity, although even if that were true, it would be unclear what the biochemical triggers of such autoimmunity would be (unmethylated RNA, absence or writer components, excess of free m6A-binding proteins etc). However, given the concerns above, I think the authors should dedicate a small paragraph in the discussion to the possibility that the primary cause of stress/defense-gene expression is unclear and may not result from innate immune surveillance of unmethylated mRNA or components of the m6A pathway as favoured by the authors.

Thank you for these very insightful comments. We did try to make a genetic test: we obtained the EDS1 CRISPR line from Jane Parker/Johannes Stuttmann, which knocks out duplicated *EDS1* genes in the Col-0 accession. However, we were unable to recover a double mutant with *vir-1*.

As we do not know the mechanism that triggers autoimmunity, we have no favoured model. We had included a paragraph in the submitted manuscript that outlined how the autoimmune response might be quite indirect, which began “It may not be the loss of mRNA…” Since we were not clear enough in communicating this, we have now revised the text in this paragraph to clarify these points in the revised manuscript.

Additionally, we have included a paragraph in the Discussion section regarding the limitations of our current understanding of Arabidopsis autoimmunity and the potential value of studying autoimmune mutants in greater detail. Early in our analysis, we had sought to compare gene expression programmes with known autoimmunity mutants, only to find that there is no signature programme of gene expression that defines autoimmunity. Indeed, we have now cited new work by Shenyang He, that we had previously overlooked, which suggests that Arabidopsis autoimmunity mutants can be grouped into at least two classes: one class, exemplified by *tip1*, depends on the presence of the leaf microbiota, while another, exemplified by *snc1*, exhibits autoimmune phenotypes that are independent of the leaf microbiota (Cheng et al., 2024 *Nat Plants* **10**, 1363-1376). Since we found that *vir-1* defence gene expression was activated at 17°C in axenic cultures, *vir-1* resembles the *snc1*-type class rather than *tip1* in this respect.

In terms of definitions, like us, Shenyang He’s most recent paper (Cheng et al., 2024) defines autoimmune mutants as “…plant mutants that show constitutively activated defence responses in the absence of pathogen infection.” I think our findings align with this definition of

autoimmunity, even if we cannot say how these defence genes are activated. Therefore, it is not clear that a genetic test is required to meet the definition of autoimmunity.

We have edited the text to avoid the idea that elevated temperature can be used as a proof of immune activation; however, in our case, what we very clearly see is immune activation at 17ºC, which is suppressed at 27ºC. Although temperature does change physiology, we (and others) do not detect such dramatic changes in defence gene expression in WT at 17ºC and 27ºC as those we detect in *vir-1*. Our study provides a previously unreleased resource on the temperature sensitivity of RNA 3' end formation and m^6A modification, utilising ONT DRS data, which has already proven to be of broader interest to the plant science community.

Finally, as is true for the autoimmune response triggered by unmodified RNAs in human cells, we have not yet established the mechanism of the trigger in this case. We do, however, present analyses of RNA processing and modification in different genetic backgrounds and temperature treatments that provide potentially valuable negative data relating to this important point.

Hopefully, the changes we have made address the reviewer's comments.

(2) It may be of relevance to search promoters of differentially expressed genes for enrichment of cis-elements. This simple approach identified the W-box in the first papers using transcriptome profiling to characterize the immune state in Arabidopsis 2,3, and could perhaps reveal whether a WRKY-driven transcriptional program drives differential expression or whether several other transcription factor classes may also contribute substantially, as may be expected if a more complex stress-related transcriptional program is activated. I do not think that this is a deal breaker, but some additional useful information from the existing data might be gathered in this way.

We are grateful for this suggestion. We searched for motifs that reflect transcription factor binding site preferences in the 1.5kb region upstream of the annotated transcription start sites of genes, which were significantly more highly expressed in *vir-1* at 17ºC compared to other conditions. Here, we use the 1.5kb region upstream of the annotated transcription start site as a simplified proxy for the promoter region, acknowledging that promoter elements may be located elsewhere. Motif enrichment was performed using AME (Analysis of Motif Enrichment) from MEME Suite version 5.5.7 and the Arabidopsis DAP V1 motif database. The 1.5 kb regions upstream of upregulated genes were compared to a background set comprising 5,000 1.5 kb regions from genes that did not show a significant increase in expression under the same conditions.

In the promoters of genes with significantly higher (logFC > 2.0, FDR < 0.001) expression in *vir-1* compared to Col-0 at 17 °C, we observed significant enrichment ($p < 0.01$) for 16 motifs. Enriched motifs included the W-box ($p = 0.0039$), as well as motifs associated with NAC, MYB, and G2-like transcription factors. At 27°C, we observe no enrichment of motifs in the promoters of genes that are significantly more highly expressed in *vir-1* compared to Col-0.

The promoters of genes significantly enriched in *vir-1* mutants at 17ºC compared to *vir-1* mutants at 27ºC were significantly enriched for multiple MYB related and G2-like binding motifs.

When comparing *vir-1* at 17 °C to all other conditions, we again found significant enrichment ($p < 0.01$) for NAC, MYB, and G2-like motifs; however, the W-box was not significantly enriched in this set. While there was no enrichment of the W-box motif in promoters of genes that had significantly higher expression in 17ºC versus other conditions, WRKY domains are significantly enriched ($FDR = 0.00038$) among the proteins encoded by these genes, as previously reported in Supplementary Table 6.

Overall, these results are consistent with the idea that not only are WRKY transcription factor-mediated gene expression changes activated in *vir-1* at 17ºC, but also a broader stress response occurs.

The summarised results of the motif search have been included in the revised manuscript, with full motif analysis (AME) included in Supplementary Table 7 (under updated numbering).

(3) Stress response activation has also been clearly described in ect2 ect3 ect4 mutants4 and even if the authors find no evidence for PR1 expression in this mutant, it is still of relevance to include a mention of this result in the discussion, together with the discussion of stress response activation seen in writer mutants in earlier reports 5,6. I would not mind the authors being a bit more explicit about what their results mean for studies that try to conclude on the biological relevance of m6A in different types of stress signaling, using phenotypes writer mutants as their primary line of evidence. But this is of course up to the authors to decide on that.

We have included the description of stress responses in the ect2,3,4 triple mutant in the Discussion section of the revised manuscript.

(4) In the introduction on preferred m6A sequence contexts, please clarify that m6A in plants occurs both DRACH in (G)GAU contexts 7,8.

We have clarified this in the Introduction section of the revised manuscript. Indeed, in earlier work, we also identified 4.8% of m^6A sites within an AGAUU motif when we first mapped m^6A using ONT DRS (Parker et al., 2020), and this is supported by the most recent analysis: Xie, YY., Zhong, ZD., Chen, HX. *et al.* Single-molecule direct RNA sequencing reveals the shaping of epitranscriptome across multiple species. *Nat Commun* **16**, 5119 (2025).

(5) When mentioning convergence on shared signaling components from immune receptors, please include a tiny bit more information for the reader. For instance, EDS1 is mentioned, but this protein is only required for signaling from (some) TIR-NBS-LRRs, not the class of CC-NBS-LRRs. Indeed, signaling by this latter class may not converge on just one to a few components, as their multimerization appears to form the ion channels required for signaling-inducing ion currents.

Thankyou for this suggestion. We have revised the text in the Introduction to avoid this error.

(6) Please clarify in the introduction and in later parts that only some forms of autoimmunity can be suppressed by elevated temperature. Sentences like "A hallmark of Arabidopsis autoimmunity is temperature sensitivity..." are a bit misleading. Temperature sensitivity has clearly been used to study some forms of EDS1-dependent immunity, to great effect in the TMV-N interaction for instance, but it is not accurate to call temperature sensitivity a "hallmark of autoimmunity".

Thank you for this insight. We have updated the text to be more precise and removed the phrase “a hallmark…” from the revised manuscript. In general, we have used the phrasing “…frequently temperature sensitive”.

(7) In the discussion of possible biochemical triggers of autoimmunity in m6A mutants, please consider the following:

(A) Mention the possibility that the primary trigger may not be immune receptor-surveillance of some defect induced by lack of m6A in mRNA (as discussed above).

We have clarified and reinforced this statement in our revised Discussion section.

(B) In connection with the consideration that lack of m6A writer components, not m6A in mRNA, may be a signal, you could include the observation from yeast that Ime4 knockouts have a much stronger phenotype than Ime4 catalytically dead mutants or knockouts of the sole yeast YTH-domain Pho92 9. Indeed, it is a bit of an embarrassment to the plant m6A community that we have not yet examined phenotypes of MTA and MTB catalytically dead mutants, and the present report should further urge the community to finally do this important experiment.

We are grateful for this suggestion, and we have included it as a new element in our revised Discussion section.

(8) Just a tiny typo on page 15, Pst DC3000, not Pst D3000 (of no relevance to the overall assessment, just a help to eliminate annoying errors before final submission).

Thank you, we have fixed this error.

REFERENCES

1. Demont, H. et al. Downstream signaling induced by several plant Toll/interleukin-1 receptor-containing immune proteins is stable at elevated temperature. Cell Reports 44(2025).
2. Petersen, M. et al. Arabidopsis MAP kinase 4 negatively regulates systemic acquired resistance. Cell 103, 1111-1120 (2000).

3. Maleck, K. et al. The transcriptome of Arabidopsis thaliana during systemic acquired resistance. Nature Genetics 26, 403-410 (2000).
4. Arribas-Hernández, L. et al. The YTHDF proteins ECT2 and ECT3 bind largely overlapping target sets and influence target mRNA abundance, not alternative polyadenylation. eLife 10, e72377 (2021).
5. Bodi, Z. et al. Adenosine Methylation in Arabidopsis mRNA is Associated with the 3' End and Reduced Levels Cause Developmental Defects. Front Plant Sci 3, 48 (2012).
6. Prall, W. et al. Pathogen-induced m6A dynamics affect plant immunity. The Plant Cell 35, 4155-4172 (2023).
7. Arribas-Hernández, L. et al. Principles of mRNA targeting via the Arabidopsis m6A-binding protein ECT2. eLife 10, e72375 (2021).
8. Wang, G. et al. Quantitative profiling of m6A at single base resolution across the life cycle of rice and Arabidopsis. Nature Communications 15, 4881 (2024).
9. Ensinck, I. et al. The yeast RNA methylation complex consists of conserved yet reconfigured components with m6A-dependent and independent roles. eLife 12, RP87860 (2023).

Reviewer #2 (Significance (Required)):

The finding that an autoimmune-like state is activated in m6A writer mutants is significant because it provides a warning flag on how such mutants should be used for studying the role of m6A in stress response signaling, including reassessment of previously published work. Whether the stress state really is autoimmunity is subject to some debate, particularly because no genetic evidence to support it has been obtained. The results are nonetheless interesting and constitute an important contribution to the community, even if they remain descriptive and with nearly no insight into molecular mechanisms. I wish to congratulate the authors on another valuable contribution to the plant m6A field.

Reviewer 3

Reviewer #3 (Evidence, reproducibility and clarity (Required)):

This study characterizes autoimmunity in mutant lines of Arabidopsis that are lacking components of the m6A methyltransferase complex (MTC). The molecular results and bacterial pathogen resistance of the lines at low temps as compared to high temps support this hypothesis. However, the phenotypic analysis or new complete lack there of (Figure 6), makes the hypothesis and overall story much less convincing. I give some comments for improving the figures below.

Figure 6 showing the phenotypes in its current set up is very uninformative. More informative pictures and quantitative analyses of specific developmental phenotypes should be added to show the differences between the phenotypes of the mutant and wild-type plants at the two different temperatures. As of now the reader gets a sense of nothing from the figure. Without this Figure demonstrating a major rescue of phenotype at the 27C temperature the reader is not convinced that the autoimmunity is the major cause of the phenotype

To clarify, we do not claim that autoimmunity is the major cause of the developmental phenotypes, but rather that the autoimmunity and developmental phenotypes are separable. We tested this by growing the plants at 17°C and 27°C, with the autoimmune mutant *acd6* included as a positive control. The data are shown in Figure 6 and Supplemental Figure 6. In contrast to *acd6*, the overall developmental differences between *vir-1* and Col-0 clearly remain at elevated temperatures. Therefore, these visible developmental defects are not rescued. In the RNA-seq snapshot we sampled, not all gene expression changes are suppressed at elevated temperatures, although the vast majority are (at the threshold levels applied in our analyses). Therefore, as we proposed in the Discussion, analysing gene expression changes by RNA-seq at other (earlier) time points in development may help reveal candidate gene expression changes that cause specific developmental changes when the mRNA m^6A writer complex is disrupted.

Supplemental Figure 1 is missing from the review file**.**

The supplemental figures are linked to each main figure. In the submitted manuscript, we did not include a supplement for Figure 1, which explains why there was no Supplemental Figure 1 and no reference to it in the manuscript text. We apologise if this organisation caused any confusion. In the revised manuscript, we have rearranged Figure 1 and relocated some elements to a new Supplemental Figure 1, as suggested by Reviewer 1.

Reviewer #3 (Significance (Required)):

It is notable that a couple of recent studies have already shown the increased resistance of MTC component mutants to pathogens (Prall et al. 2024 and Chen et al. 2024), which weakens the

impact of the overall findings. In my honest assessment, this study would be well positioned for publication in a mid-tier plant specific journal (e.g. Plant Physiology) based on the currently included results.

To clarify, Prall et al., 2023, did observe increased pathogen resistance in different mRNA m^6A writer mutants, including the *fip37-4* allele that we report here. However, Chen et al., 2024 did not report this, despite also using *fip37-4*. I think this highlights an important implication of our work, as outlined in the Discussion section, that the environmental sensitivity of mRNA m^6A writer complex mutants necessitates the control of environmental treatments or the amelioration of autoimmunity to study the role of mRNA m^6A in a meaningful manner. The major conceptual advance we make here is that disruption of the mRNA m^6A writer complex triggers autoimmunity. Neither of these recent studies makes this point, but the recognition of the widespread indirect gene expression changes that flow from autoimmunity compromises the ability to dissect the direct role of mRNA m^6A in perhaps all aspects of plant biology.
